# Growing resilience capacity for learners presenting with specific learning disability in learners with special education needs schools

**DOI:** 10.4102/ajod.v12i0.1045

**Published:** 2023-04-18

**Authors:** Daphney Mawila

**Affiliations:** 1 Department of Educational Psychology, Faculty of Education, University of Johannesburg, Johannesburg, South Africa

**Keywords:** individual factors, contextual factors, learners with special education needs, relationships, resilience, resources, risk, specific learning disability

## Abstract

**Background:**

Preventing adversity from accelerating among learners with specific learning disabilities (SLD) is imperative. Continuous adversities, such as social-emotional, psychological and academic difficulties, characterise learners with SLD. Prior studies have been conducted on learners with SLD developing a disorder because of the difficulties they face. However, very few studies offer evidence of how learners presenting with SLD cope despite their learning disability.

**Objectives:**

The study sought to investigate what resilience resources are available among learners with SLD in learners with special education needs (LSEN) schools and to provide stakeholders with evidence of resilience enablers for learners with SLD.

**Method:**

An exploratory quantitative research study was adopted, and 217 respondents with SLD were selected through purposive sampling in four LSEN schools. These learners completed the Child and Youth Resilience Measure (CYRM-28). Data were analysed using the Statistical Package for the Social Sciences (SPSS) and the custom table was used as a statistical technique

**Results:**

Even though the presence of SLD negatively affects an individual’s academic, psychological, social and emotional functioning, the results of this study show that individual qualities, relationships with caregivers and peers and contextual resources were resilience-enabling resources for learners with SLD.

**Conclusion:**

The study’s results show that the combination of individual attributes, relational and environmental factors enables the resilience of learners with SLD. When given accessible and meaningful support, learners with SLD can develop resilience.

**Contribution:**

The study contributes to the dearth of knowledge regarding the resilience of learners with SLD in LSEN schools.

## Introduction

Specific learning disabilities (SLDs) result from biological and contextual factors. The Diagnostic and Statistical Manual, 5th Edition (DSM-5) (2013) defines SLD as one of the neurodevelopmental disorders that originate from biology and comprise the interaction of genetics and contextual factors. The interaction between these factors affects the ability of the individual to process information adequately. Specific learning disabilities include disabilities in basic education skills like reading, listening, speaking, written expression or mathematical calculations (Grigorenko et al. 2020). Children with SLDs often suffer continuous adversities, such as academic problems, which have implications on their learning and the functioning of their families. The challenges faced by learners with SLDs extend to their social, emotional and psychological functioning. Specific learning disabilities could result in repeated failures in school and dropping out. Learners who drop out of school are likely to have adverse consequences on their quality of life, increasing the risk of relying on social welfare organisations (Kohli, Sharma & Padhy 2018). Thus, SLDs cause stress to learners’ lives, both at school and afterwards. Norris et al. (2020) argue that the social and emotional problems faced by learners with SLDs are likely to continue into adulthood and limit their prospects of success. The combination of a stressful life and everyday difficulties could result in nonresilient outcomes for learners with SLDs.

Researchers (see Haroardottir, Juliusdottir & Guomundsson 2015; Morrison & Gosden 2019) state that SLD is a risk factor as it predicts both affirmative and undesirable outcomes. Many children with SLD develop disorders due to the inability to resile in the face of hardships in their lives. However, some individuals with SLD manage to cope with adversities despite the presence of SLD in their lives and are resilient (Panicker & Chelliah 2016). Ungar (2019) states that resilience is evident when an individual faces adversity. In prior studies, researchers found successful, well-adjusted individuals, despite the hardships faced by learners with SLD (Haroardottir et al. 2015). A study by Holtge et al. (2021) states that there are factors within learners’ social ecology that could enhance their resilience and assist them in overcoming the challenges accompanying the presence of SLD. Ebersöhn (2019) points out that resilience mitigates risk factors impacting individuals’ functioning. Resilience is defined as:

[*T*]he process of negotiating, managing and adapting to significant sources of stress or trauma. Assets and resources within the individual, their life and environment facilitate this capacity for adaptation and bouncing back in the face of adversity. (Windle 2011:163)

Masten (2014) affirms that resilience is an individual journey that anyone can develop through their thoughts and behaviour. To bounce back from adversities, a person needs to develop effective coping techniques. Masten regards resilience as a person’s ability to do well in the face of adversities. Ungar (2013) agrees that doing well refers to individuals who display positive development, despite the adversity that would usually predict adverse consequences. Thus, in this study, the author argues that resilience resources enhance the resilience of learners presenting with SLD. This was confirmed in a study by Windle, Bennett and MacLeod (2020), where the accessibility of resources facilitated resilience. Because of the high prevalence of SLD in learners (Grigorenko et al. 2020) and its associated negative impact on the overall functioning of individuals (Kohli et al. 2018; Norris et al. 2020), the current study looked closely at what enables them to cope despite SLD. Several studies (see Fletcher et al. 2018; Seidenberg 2017) have been conducted that describe SLD, its causes and diagnosis, as well as how it affects learners’ learning. Studies on resilience (see Panzeri et al. 2021; Van Breda & Theron 2018) have also been conducted on various topics but very few on the resilience of learners with SLD. Therefore, this study investigated the resilience of learners with SLD in learners with special education needs (LSEN) schools to add to the growing body of knowledge on this topic and to provide a foundation for future studies and interventions focused on building resilience in learners with SLD.

Scientifically, researchers in African and non-Western continents are faced with the challenge of decolonising psychology to their specific contexts and population. Theron (2013) paid attention to cultural resilience indicators in South Africa. Theron and Phasha (2015) state that:

[*M*]any of the resilience processes identified in international studies manifest locally also, including attachment, mastery, and meaning making. However, these authors note that South African studies also focus on indigenous worldviews, including spirituality, a duty of kin and individuals’ interdependence. (p. 54)

For Africans, attachments extend to extended family members, not mainly to parents (Theron & Theron 2010). In this case, practitioners working with learners with SLD need to consider their resilience based on their context and indigenous worldviews. This study is informed by, and in alignment with, a social-ecological perspective of resilience.

### A social-ecological framework of resilience

This article is part of a larger scope of the author’s doctoral thesis, which was informed by Ungar’s social-ecological framework of resilience. This framework of resilience’s origin can be traced from Bronfenbrenner’s bio-ecological system theory (Bronfenbrenner 1974). It suggests that the context has a pivotal role in individuals’ development and provides insights into why individuals bounce back from adversity. A child’s resilience depends on the individual and contextual factors that interact together. Lessard et al. (2014) assert that resilience resources for learners with SLD include intrinsic individual abilities and external contextual support (such as parents, peers, school, neighbourhoods and communities) that diminish the probability of adversities. Theron (2016) argues that there is a need to comprehend what allows certain learners with SLD to resile but not their counterparts with similar obstacles. Up to the time of writing, the question about what enables learners with SLD to cope with their learning disability remained unanswered, as there are few studies that have focused on SLD and resilience. For this reason, the study was conducted to provide some insights into the resilience profile of learners presenting with SLD in the LSEN school environment.

### Aim and objectives

This current study aimed to investigate the resilience resources among learners with SLD in LSEN schools in Gauteng. To achieve this, the study sought to explore which individual factors, relational and contextual factors are available to learners with SLD and enable their resilience despite their learning disabilities.

## Research methods and design

This article is one of the outcomes of the researcher’s doctoral study. The main aim was ‘to investigate the relationship between social-ecological support and resilience among learners with SLD in LSEN schools’ (Mawila 2019:iv).

The study adopted an exploratory quantitative design to explore factors that enable the resilience of learners presenting with SLD. A total of 217 learners with SLD in four LSEN schools in the greater Johannesburg area in the Gauteng province, South Africa, took part in the study. Only respondents who showed a willingness to take part in this study were selected. Male and female respondents who were aged 10–19 years were purposefully selected from LSEN schools: one on the West Rand, one in Johannesburg North, one in Soweto and one in Elspark. These schools were chosen for this study because they are LSEN schools for learners with SLD. The researcher requested authorisation from school principals to collect data for a larger-scale doctorate study. The researcher also discussed this with caregivers and their children during the study.

Data was collected quantitatively, using the Child and Youth Resilience Measure (CYRM-28) (Liebenberg, Ungar & Van De Vijver 2012). The CYRM-28 intends to provide ‘a more comprehensive understanding of the processes of resilience across culture and context, accounting for the heterogeneity of culture and experiences of youth’. (Liebenberg et al. 2012:87). The categories of the CYRM-28 are based on the individual, relationship and community (culture, spirituality and education) resilience resources. Liebenberg, Ungar and LeBlanc (2013) indicate that the CYRM-28 is:

[*A*] self-report instrument validated originally with a purposive sample of 1451 youth growing up and facing diverse forms of adversity in 11 countries (Canada, USA, Colombia, China, India, Russia, Palestine, Israel, Tanzania, the Gambia, and South Africa). (p. 131)

Three student psychologists at the University of Johannesburg’s educational psychology department were employed as research assistants in this study. Only multilingual research assistants were selected to ensure that they were able to speak the languages spoken by respondents in the four schools (this included Setswana, Afrikaans, English, IsiXhosa, Xitsonga, Sesotho and IsiZulu languages). The researcher discussed with the research assistants the purpose of the study, and training of the research assistants was done by the researcher. The researcher and research assistants translated the CYRM-28 items from English to local languages; however, back translation was not done. The assistants’ role was to administer the questionnaires; this included reading, translating, monitoring and assisting respondents in completing the items. Because of the nature of the study and some respondents’ inability to read due to SLD, research assistants were sought to read the items of the CYRM-28 to ensure a precise understanding of items. Translations of items to other languages were done by research assistants if required. Translation of the CYRM-28 into another language was also done in a study by Ungar and Liebenberg (2011) to ensure accuracy.

To analyse data, the software popularly known as the SPSS (Statistical Package for the Social Science) version 25 (IBM Corporation, Armonk, New York, United States) was used. The researcher used an SPSS statistical technique called the custom table, developed by IBM SPSS, which summarises ordinal data. The services of the University of Johannesburg Statkon (statistics consulting company) Department were sought for data analysis assistance.

### Ethical considerations

Firstly, permission to conduct this study was granted by the Faculty of Education Ethics Committee (reference number Sem 2 2018-007) at the University of Johannesburg, followed by the Gauteng Department of Education (GDE). Secondly, the Resilience Research Centre^1^ also approved the researcher’s use of the CYRM-28 questionnaire in this study. Thirdly, the school headmasters further permitted the researcher to conduct research in their schools and to collect data. Thereafter, caregivers (parents or legal guardians) of learners were informed about the study and its purpose. They were also provided information on how their children would participate in the study and how the completed questionnaires would be protected and kept confidential. Lastly, once caregivers signed consent forms, learners as respondents of the study also signed the assent form (10–12 years) or consent form (13 and above) to agree with their willingness to participate in this study. During data collection and as per the requirement in a quantitative study, respondents were informed that they should not write their names on the questionnaires to ensure anonymity. They were also informed that they could withdraw from the study and that no action would be taken against them if they did. They were also told that an educational psychologist was on standby during data collection should they need someone to talk to. Respondents were informed of the South African Depression and Anxiety Group (SADAG) toll-free number in case they needed psychological assistance after the study was concluded.

## Results

Before data was collected, the researcher sought the services of a statistician at the University of Johannesburg’s Statkon Department to discuss the study’s purpose and assess the validity and reliability of the measure in this specific study. Based on the study’s objectives, the statistician advised on the best statistical techniques to use. Once this was done, data were collected and the researcher captured data on the SPSS version. Thereafter, the statistician completed the data analysis and a meeting was held to discuss the analysis. The results were then e-mailed to the researcher for write up.

Table 1, Table 2 and Table 3 show the percentages, means, frequency count and standard deviations for the CYRM-28 primary factors, such as individual, relationship and context. A Likert scale comprising five points was used to measure the responses of the CYRM-28. However, the upper and lower scales were joint for straightforward interpretation as advised by Morgan et al. (2007). For this specific study, the availability of the resources that enable the resilience of learners presenting with SLD was assessed using the custom table. Results of the study were categorised into the individual, relationships with others and contextual domains.

**TABLE 1:** Individual factors.

Items	Not at all or a little		Somewhat		A great deal or quite a bit		Mean	Std. dev.
	Count	Row *N* %	Count	Row *N* %	Count	Row *N* %		
I cooperate with people around me.	48	22.2	31	14.3	138	63.6	3.63	1.281
I try to finish what I start.	25	11.5	26	12.0	166	76.5	4.16	1.124
I am able to solve problems without harming myself or others (for example, by using drugs and/or being violent).	51	23.5	22	10.1	144	66.4	4.17	1.136
People think that I am fun to be with.	20	9.2	28	12.9	169	77.9	3.82	1.476
I am aware of my own strengths.	15	6.9	18	8.3	184	84.8	4.34	1.029

*Source*: Mawila, D., 2019, ‘Relationship between resilience and social ecological support among learners with specific learning disability in LSEN schools’, PhD thesis, University of Johannesburg

**TABLE 2:** Relationships.

Items	Not at all or a little		Somewhat		A great deal or quite a bit		Mean	Std. dev.
	Count	Row *N* %	Count	Row *N* %	Count	Row *N* %		
My parent(s) or caregiver(s) watch me closely.	16	7.4	16	7.4	185	85.3	4.42	0.974
If I am hungry, there is enough to eat.	13	6.0	20	9.2	184	84.8	4.44	0.980
I talk to my family or caregiver(s) about how I feel.	78	35.9	36	16.6	103	47.5	3.24	1.483
My family stands by me during difficult times.	20	9.2	24	11.1	173	79.7	4.33	1.040
I feel safe when I am with my family or caregiver(s).	6	2.8	18	8.3	193	88.9	4.62	0.825
My parent(s) or caregiver(s) know a lot about me.	31	14.3	20	9.2	166	76.5	4.17	1.186
I enjoy my family’s or caregiver’s cultural and family traditions.	18	8.3	23	10.6	176	81.1	4.36	1.067
I feel supported by my friends.	33	22.5	30	13.8	154	71	4.00	1.260
My friends stand by me during difficult times.	28	12.9	23	10.6	166	76.5	4.15	1.169
I know how to behave in different social situations.	45	20.8	25	11.5	147	67.7	3.83	1.292
I have opportunities to show others that I am becoming an adult and can act responsibly.	16	7.4	26	12.0	175	80.6	4.30	1.031
I have opportunities to develop skills that will be useful later in life (like job skills and skills to care for others).	10	4.6	8	3.7	199	91.7	4.55	0.827
I know where to go in my community to get help.	48	21.2	24	11.1	145	66.8	3.76	1.481

*Source:* Mawila, D., 2019, ‘Relationship between resilience and social ecological support among learners with specific learning disability in LSEN schools’, PhD thesis, University of Johannesburg

**TABLE 3:** Context factors.

Items	Not at all or a little		Somewhat		A great deal or quite a bit		Mean	Std. dev.
	Count	Row *N* %	Count	Row *N* %	Count	Row *N* %		
I think it is important to serve my community.	26	12.0	29	13.4	162	74.7	4.11	1.156
Spiritual beliefs are a source of strength for me.	24	11.1	23	10.6	170	78.3	4.26	1.150
I participate in organised religious activities.	55	25.3	30	13.8	132	60.8	3.69	1.457
I feel I belong at my school.	26	12.0	28	12.9	163	75.1	4.15	1.257
Getting an education is important to me.	5	2.3	9	4.1	203	93.5	4.69	0.702
I am proud of my ethnic background.	18	8.3	20	9.2	179	82.5	4.45	1.049
I am treated fairly in my community.	19	8.8	32	14.7	166	76.5	4.18	1.104
I have people I look up to.	19	8.8	18	8.3	180	82.9	4.37	1.098
I enjoy my community’s traditions.	46	21.2	59	27.2	112	51.6	3.69	1.405

*Source*: Mawila, D., 2019, ‘Relationship between resilience and social ecological support among learners with specific learning disability in LSEN schools’, PhD thesis, University of Johannesburg

### Individual domain

Individual resilience factors refer to assets, qualities and strengths a person possesses which enable them to combat their adversities. Table 1 illustrates the custom table for individual factors.

As evident by the high mean values ranging between 3.63 and 4.34 in Table 1 (individual factors), respondents were conscious of their abilities. The CYRM-28 revealed that individual factors enable the resilience of learners with SLD. Table 1 displays that self-efficacy was critical to learners with SLD; 166 respondents reported finishing what they started because they have faith in their competencies. Learners with SLD also viewed self-awareness as imperative for the development of resilience. The results on the CYRM-28 displayed that learners know their strengths, as revealed by 184 learners reporting awareness of their inner strengths. Thus, self-awareness was a resilience resource regardless of the presence of SLD. In addition, the ability to problem solve was viewed as enabling them to cope regardless of SLD. This study demonstrated that 144 respondents stated that they could solve problems effectively without hurting themselves and others. This shows that their problem-solving ability enhanced their resilience. Lastly, self-confidence was noticed as resilience enabling; 169 respondents stipulated that people view them as fun to be with, suggesting that confidence in themselves makes others happy.

### Relationship domain

Meaningful relationships are critical in enabling individuals to reinforce resilience. This study found that learners with SLD viewed relationships with caregivers and friends as resilience-enabling resources. Table 2 illustrates the custom table for relationships.

The variables observed in Table 2 (relationships) show that items assessing relationships demonstrated affirmative responses, with mean scores above three. Learners with SLD reported that their families care for their psychological and physical needs. The relationships they have with their caregivers enable their resilience. A total number of 185 learners recognised that their caregivers pay close attention to them, and 184 testified they have sufficient food to eat when hungry. Moreover, 193 respondents felt safe around their family or caregivers, and 173 said their family members supported them during challenging times. In this study, meaningful interactions with peers emerged as resilience factors for learners presenting with SLD. Child and Youth Resilience Measure results revealed that 166 learners conveyed that their friends stand by them in trying times and 154 of them felt supported by their friends. Thus, positive connections and attachments with peers are imperative for learners with SLD to combat their challenges with confidence.

Furthermore, the study revealed that social competence is a resilience enabler. Social competence refers to a person’s ability to engage and relate to others positively (Junge et al. 2020). A significant number of the respondents in this study reported having social competence skills, as 199 respondents attested to having opportunities to advance skills valuable for their future. In comparison, 175 respondents confirmed that they have a chance to show people that they can act responsibly and become adults. In this study, interacting with other people emerged as a resilience resource.

### Context domain

Context is an important resilience resource, and the treatment the individual receives from their context plays a significant role in their resilience development. Table 3 presents the custom table for items measuring respondents’ responses to contextual factors.

The above three mean scores in Table 3 (context factors) proposed that learners are conscious of contextual opportunities available to them. In this study, 166 respondents reported being well treated in their community. Respondents considered spirituality as enabling resilience; 170 respondents perceived spiritual beliefs as a basis of strength. The results of this study imply that learners knew of accessible spiritual opportunities in their environments. Serving the community is a resilience enabler for learners with SLD; 162 learners regarded it as necessary to serve their community. Contributing to a community in this study was viewed as resilience-enabling, as it gives learners with SLD a sense of determination and purpose in life.

Moreover, education is regarded as important to learners with SLD and is a resilience resource, as 203 learners regarded education as of fundamental value. One of the critical resilience enablers in this study was the respondents’ sense of belonging at their schools. Many respondents (163) reported feelings of belonging in their school. Respondents in this study are in an LSEN school; a sense of belonging could be attributed to the schools being designed to meet their educational needs. Furthermore, culture was also regarded as an enabler of resilience. The CYRM displayed that 180 learners have access to individuals they look up to in different cultures and 179 learners stated being proud of their ethnic upbringing. The study results recommend that accessible role models enable the resilience of SLD learners in their respective cultures. Thus, the positive influence of role models gives them an aim to aspire to and supports learners in coping with adversities that come with having SLD.

## Discussion

The CYRM-28 was used in this study to collect quantitative data to explore resources contributing to the resilience of learners presenting with SLD in LSEN school contexts. Three domains were established based on the results of the CYRM-28, namely individual factors, relationships and context.

Key results of the study show that respondents’ individual attributes, such as problem-solving, self-confidence, self-efficacy and self-awareness, enable them to overcome adversities. Self-efficacy was found in the CYRM-28 questionnaire as essential for learners presenting with SLD in LSEN schools. A prior study by Amitay and Gumpel (2015) noted that self-efficacy emerged as a resilience factor among schoolgirls with SLD. Álvarez et al. (2022) also found that self-efficacy enabled adolescents to cope with adversities. Respondents of this study acknowledged awareness of their personal strengths in the CYRM-28. Similarly, a study by Martins and Neto (2016) found that self-awareness was a significant protective factor in promoting people’s resilience. For learners with SLD, self-awareness and problem-solving ability were critical to bouncing back when faced with SLD adversities, thus enabling their resilience. In correlation with this study, Malindi’s study on street children also revealed that problem-solving ability was resilience enabling (2014), and this links to the individual traits explained in the social ecology framework of resilience (Ungar 2019). Furthermore, self-confidence was seen as enabling resilience in this study, which was consistent with Malindi’s finding that self-confidence was a resilience resource for street adolescents. Thus, previous studies correspond with the current study’s results, which found that certain individual traits are evident resources for resilience.

In the relationship domain, the factors that enabled resilience for learners with SLD were relationships with parents or caregivers, meaningful relationships with friends or peers and social competence. Positive interactions with other individuals emerged as a resilience factor. Luthar, Ebbert and Kumar (2021) state that parent–child attachments enhanced and fostered children’s resilience during the coronavirus disease 2019 (COVID-19) pandemic. Enabling resilience factors such as supportive relationships with others are part of the family processes at the microsystem level. Recent literature confirms that these relationships buffer stressful life situations (Bellis et al. 2017; Van Rensburg, Theron & Rothmann 2015). For learners with SLD in South Africa, it should be considered attachments with others, including significant people in their immediate and extended family, neighbours and other community members. This aligns with a popular African proverb, ‘it takes a village to raise a child’. Mikucka and Rizzi (2016) assert that this proverb implies that the whole community has a responsibility in raising children, and the responsibility does not only lie on the parental shoulders. Sefotho (2019) affirms that community members are committed to supporting their individuals. In African contexts, community members are expected to support and provide individuals with resources to enable their development and functioning.

For individuals to bolster resilience, meaningful attachments are critical. In this study, learners with SLD reported that meaningful relationships with friends and peers were resilience enabling and assisted them in combating SLD with self-confidence. A study by Graber et al. (2016) found similar results. Their study found that connections and friends promote resilience for individuals in South Africa because they enable communication, social acceptance, support for each other and positive interaction. Therefore, these friendships are critical in providing learners with SLD with valuable emotional support. Studies (see Theron 2014; Van Harmelen et al. 2017) also state that friendships among individuals facing adversity foster adjustment, flexibility and healthy development.

The study further revealed that social competence was also viewed as a resilience resource. Social competence is a fundamental skill for relating to other people and is critical in developing relationships. According to Saito and Okayaso (2014), social competency is linked to resilience and is the basis for creating relationships with other people. A study by Gómez-López et al. (2022) also revealed that social competence among adolescents was resilience enabling.

Within the context domain, the CYRM-28 results further revealed that learners with SLD considered having access to resources within their context as a factor enabling their resilience. These resources include access to education, spirituality, role models, serving the community and cultural identification. A study by Malindi (2014) stipulated that for young people to be resilient, access to school facilities, education and information is imperative. The study corroborates prior studies. For example, Theron (2016) revealed that Sesotho-speaking individuals regard education as crucial because it offers socio-economic advantages for their lives, families and community. Other resilience studies (see Theron 2017; Van Breda 2017) also reported the significance of education as a resilience enabler for South African black youth. In addition, a study by Van Rensburg et al. (2013) noted that southern Sesotho-speaking adolescents perceived education as supportive to their resilience.

Furthermore, a sense of belonging at LSEN schools was also found to be resilience enabling for learners with SLD in this study. A Turkish study revealed that students’ positive feelings towards school and a sense of belonging at school were factors that enabled resilience (Yilmaz 2016). A sense of belonging encourages positive development in youth, empowering them to utilise the resources within their community. The study also found that spirituality for learners with SLD assisted them in combating adversities. This result correlated with a study by Young (2017), which affirmed that faith was the source of resilience for their participants. However, an interdisciplinary approach is needed when issues of spirituality are addressed because human beings are complex (Vinueza 2017).

Moreover, the study’s results further suggest that the availability of role models for learners’ respective cultures was a vital resilience resource. Leaners presenting with SLD overcome adversities due to their role models’ aspiration and positive influence. The study corresponds with previous studies indicating that having a role model enables resilience. To illustrate, a study by Malindi (2014) on street children reported that having role models contributed to their resilience, regardless of adverse circumstances they found themselves in. Additionally, Abaza and Nelson (2018) also found that role models enabled the resilience of learners.

Additionally, a sense of duty was also viewed as a significant resilience factor for learners with SLD, because individuals have a role to play in improving the world. In this study, learners with SLD reported that serving their community enabled their resilience. A sense of duty towards the community links to ideologies of the viewpoint of *ubuntu. Ubuntu* refers to the idea that ‘a person is only a person through other persons’ (Tutu 2006:122). Subsequently, learners viewed their role in participating for the betterment of their communities, thus assuming the role of agents of change. The results of the study further confirmed what the social-ecological framework of resilience maintains, namely that learners’ resilience is a combination of individual factors, relationships with others and contextual resources. Cinner and Barnes (2019) affirm that these codependent connections between individuals and ecologies build resilience. It is the interdependence of social-ecological resources that accounts for the resilience of learners with SLD despite their learning disabilities. In light of this, this study challenges the view that learners with SLD are not resilient and that they are prone to become dropouts, as some studies have previously suggested (see Grigorenko et al. 2020; Norris et al. 2020).

### Strengths and limitations

The study provides insights into the resilience coping mechanisms of learners presenting with SLD. This study contributes to the dearth of literature on the resilience of learners with SLD in LSEN contexts. The study’s results could be a foundation for developing primary interventions for learners presenting with SLD in LSEN schools and adequately overcoming adversity before resulting in pathology. Consequently, this could reduce the high dropout rate in schools. The current study advocates that parents, schools and community members have a pivotal role in championing learners’ resilience. Collaboration between stakeholders is imperative for fostering their resilience despite SLD.

The study had several limitations. A self-report questionnaire was used to collect data, and respondents may have responded to items based on construct biases and the likelihood of the items. Additionally, respondents’ completion of the CYRM-28 could have been affected by their experiences and emotions at the time of completing the measure. Despite this, Haeffel and Howard (2010) stress that self-report measures provide valued insights into the constructs studied. To ensure a fair comprehension of the CYRM-28 items, research assistants translated items for learners in an LSEN school in Soweto. Translating items from English to different home languages extended the data collection period, as some words were not possible to translate, which may have been detrimental to the reliability of the CYRM-28 questionnaire.

## Conclusion

This study establishes a foundation for future research to be conducted in a similar context, namely to investigate the resilience resources that enable learners with SLD in LSEN schools to cope with their learning disabilities. The CYRM-28 results confirm that learners with SLD are not necessarily as vulnerable as previously thought; they can use the resources in their environment to enable their resilience. In the absence of risks, resilience is nonexistent and individuals’ adaptation processes to alleviate risks are not noticeable. Studies on resilience have highlighted various risks and resilience processes. Nevertheless, minimal studies have focused on learners presenting with SLD in LSEN schools and their resilience. Thus, this study investigated the resilience resources of learners with SLD in LSEN schools.

This study calls upon parents, school personnel, mental health experts and scholars to consider the factors that enable resilience when dealing with learners with SLD. The study concludes that researchers, teachers, psychologists, parents and community members have a duty to make available resilience resources for learners presenting with SLD in LSEN schools. In contrast, learners have a role in making better use of accessible resources. The results of this study apply only to LSEN schools. It would be worthwhile to gain some insights into the resilience factors of learners presenting with SLD in full-service or mainstream schools. Further studies could include qualitative measures to explore how the resources identified are used by learners with SLD to enable their resilience. Further research is required regarding interventions to promote learners’ resilience for successful development and application. During the COVID-19 pandemic, learners with SLD could not attend school for an extended period, and some had to use technology to assist their learning. Future studies need to consider the impact of the COVID-19 pandemic on the resilience of the respondents, using quantitative and qualitative methods.

## Footnotes

1
http://CYRM-28.resilienceresearch.org/
